# Radiation-induced Undifferentiated Malignant Pituitary Tumor After 5 Years of Treatment for Cushing Disease

**DOI:** 10.1210/jcemcr/luad119

**Published:** 2023-11-06

**Authors:** Gazal Bakshi, Sunil Kumar Mishra, Vishnupriya AR, Virendera Pal Singh

**Affiliations:** Department of Endocrinology and Diabetes, Medanta, The Medicity, Gurugram, 122001, India; Department of Endocrinology and Diabetes, Medanta, The Medicity, Gurugram, 122001, India; Department of Endocrinology and Diabetes, Medanta, The Medicity, Gurugram, 122001, India; Department of Endocrinology and Diabetes, Medanta, The Medicity, Gurugram, 122001, India

**Keywords:** radiotherapy, second malignancy, Cushing disease

## Abstract

The occurrence of a second neoplasm possibly constitutes an adverse and uncommon complication after radiotherapy. The incidence of a second pituitary tumor in patients irradiated for adrenocorticotropic hormone secreting pituitary adenoma is rare. We report a case of a 40-year-old female with Cushing disease who underwent surgical management followed by radiotherapy. After 5 years of initial treatment, an increase in tumor size was evident at the same location, with a significant interval growth of the parasellar component of the lesion. Histology revealed an undifferentiated highly malignant sarcoma. In the span of next 2 years, the patient was followed with 2 repeat decompression surgeries and radiotherapy because of significant recurrent compressive symptoms by locally invasive malignant tumor. Despite the best efforts, the patient remained unresponsive to multiple treatment strategies (eg, surgical resections and radiotherapy) and succumbed to death.

## Introduction

Radiation therapy is a commonly used modality for primary or adjuvant treatment of pituitary adenoma. It is also used as an adjuvant therapy for Cushing disease with persistent or aggressive tumor growth or recurrent disease after surgery. The immediate sequelae of radiotherapy for pituitary tumors include nausea, fatigue, diminished taste and olfaction, and hair loss [1]. One frequent long-term side effect is hypopituitarism. The incidence rate of new-onset hypopituitarism after conventional radiotherapy is approximately 30% to 100% after a follow-up of 10 years, whereas after stereotactic radiosurgery or fractionated radiotherapy, the incidence is approximately 10% to 40% at 5 years [2].

The occurrence of a second neoplasm after cranial radiotherapy constitutes possibly one of the most adverse complications. Tumors such as meningioma, glioma, and sarcoma are the most frequently reported secondary neoplasms after pituitary irradiation [3]. The cumulative probability of a second brain tumor in patients irradiated for pituitary adenoma and craniopharyngioma is approximately 4% [4].

We report 1 such case with detailed clinical, histopathological, and radiological characteristics because of its rarity and associated high mortality of radiation-induced sarcoma.

## Case Presentation

The patient first presented at 40 years of age with complaints of weight gain, new-onset diabetes mellitus, hypertension, and cushingoid features in 2014. She was diagnosed with Cushing disease (24-hour urinary cortisol 1384 mcg/24 hours [3819 nmol/24 hours; reference >2 upper limit of normal], low-dose dexamethasone suppression test serum cortisol 16.6 mcg/dL [457.9 nmol/L], ACTH 85 pg/mL [18.7 pmol/L; reference range, <46 pg/mL, <10 pmol/L]) caused by invasive adrenocorticotropic hormone-secreting giant adenoma. The initial imaging revealed a homogenously enhanced pituitary macroadenoma with a size of 42 × 37 × 35 mm with suprasellar extension and encasing both the internal carotid arteries with mass effect on optic chiasma and sellar erosion. The patient underwent tumor excision by endoscopic transsphenoidal transnasal approach. Partial excision of the tumor was achieved because of cavernous sinus invasion. Histopathology and immunohistochemical stains demonstrated a corticotrophin-secreting (ACTH-staining positive) pituitary adenoma with MIB labeling index of 1% to 2%. Because biochemical remission was not achieved (urinary cortisol 794 mcg/24 hours [2191 nmol/24 hours]; ACTH 66 pg/mL [14.5 pmol/L; reference range, <46 pg/mL, <10 pmol/L]), the patient was started on ketoconazole and was received fractionated radiotherapy with a dose of 5040 cGy in 28 fractions.

## Diagnostic Assessment

For the next 5 years, at yearly follow-up, 400 mg ketoconazole was continued in view of insufficient control of ACTH secretion. During follow-up, the size of the tumor was stable at approximately 23 × 16 × 33 mm after radiotherapy with no significant clinical and biochemical changes.

Five years after surgery and radiotherapy, the patient developed cerebrospinal fluid rhinorrhea; imaging revealed a cystic transformation of the suprasellar component and increase in the size of the tumor to 39 × 22 × 26 mm, which included visualization of a parasellar component of size 29 × 19 × 15 mm. The patient continued on ketoconazole. The patient was also advised to undergo hypofractionated radiotherapy but did not return for follow-up.

## Treatment

In 2021, 1.5 years after the last visit, the patient developed severe headache, altered sensorium, ptosis, focal seizures, and left-sided hemiparesis. During this episode, the patient had an ACTH of 66 pg/mL (14.53 pmol/L; reference range, <46 pg/mL [<10 pmol/L]) and baseline cortisol of 25 mcg/dL (689 nmol/L; reference range, 4-18 mcg/dL [110-496 nmol/L]). Repeat imaging revealed a significant decrease in the suprasellar cystic component but an increase in the size of the parasellar component to 38 × 21 × 25 mm from 29 × 19 × 15 mm, which was isointense on T1 and T2 with heterogeneous enhancement. Significant brain stem compression and perilesional edema was also visible. The patient underwent urgent frontotemporal craniotomy and decompression of the tumor. On pathological examination, the tumor tissue was composed of small pleomorphic round cells arranged in sheets and cords separated by delicate fibrocollagenous stroma. Cells had a round to oval hyperchromatic nucleus with scanty cytoplasm. Areas of hemorrhage, necrosis, and a few apoptotic bodies were seen. The tumor tissue had very high mitotic activity of >10/10 hpf and MIB labeling index of 70%. Immunohistochemistry demonstrated positivity for vimentin, CD99, and TLE-1. Dot-like positivity was present for HMB 45, synaptophysin. INI-1 loss was present in some cells. Ten percent patchy positivity was present for p53. The tumor cells, however, consistently failed to express smooth muscle actin, CD34, Myf-4, epithelial membrane antigen, desmin, LCA, SADD4, CD138, and S-100 protein. ACTH and staining for other hormones was negative. Based on the immunological and histochemical patterns, a diagnosis of high-grade poorly differentiated malignant tumor with a probability of undifferentiated sarcoma was made.

**Figure 1.**
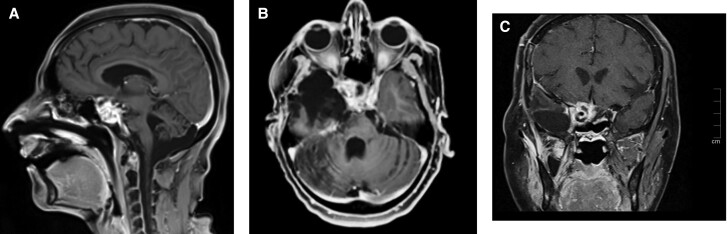
Contrast-enhanced T1 magnetic resonance imaging dynamic pituitary scan (A, sagittal; B, axial; C, coronal sections) reveals postoperative changes with residual enhancing tumor in the right lateral sella cavity with extension into the right cavernous sinus and parasellar region encasing the cavernous and inferiorly extends through the foramen ovale below the skull base up to approximately 1.5 cm. Anteriorly, it extends up to the right orbital apex and posteriorly extends along the right dorsal surface of clivus.

Because of the invasion of surrounding structures and surgical inaccessibility, repeat fractionated radiotherapy was given with a dose of 4500 cGy over 25 fractions at 1.8 Gy daily to the planned target volume via image-guided fractionated radiotherapy. During the next 1.5 years, patient improved clinically with no significant increase in the size of tumor (Fig. 1). The patient was gradually tapered from ketoconazole and developed hypopituitarism requiring levothyroxine and glucocorticoid replacement. There was a significant improvement in the power of the left side and ptosis.

## Outcome and Follow-up

**Figure 2.**
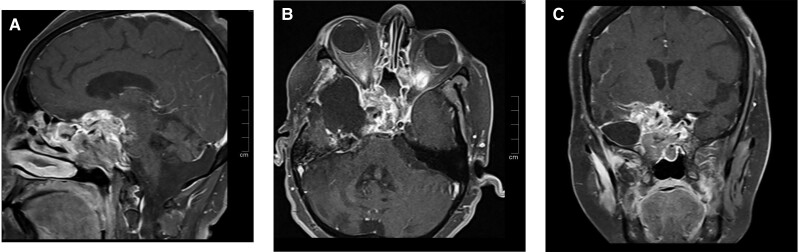
Contrast-enhanced T1 magnetic resonance imaging dynamic pituitary images (A, sagittal; B, axial; C, coronal sections) after 1.5 years of a second session of radiotherapy reveal a significant interval increase in size of heterogeneously enhancing irregular soft tissue in sellar cavity with extension into the right cavernous sinus and parasellar region when compared with previous imaging. Superiorly, it extends in the suprasellar region, causing mass effect on the optic chiasma with encasement of the right prechiasmatic optic nerve and right-sided optic chiasma. Inferiorly, the lesion extends into the sphenoid sinus. Posteriorly, there is interval increase in the lesion involving the clivus and extending into the prepontine and interpeduncular cistern. Anteriorly, mass has reached up to the right orbital apex optic nerve canal, which shows mild interval increase.

After 1.5 years of reradiation in 2022, the patient again developed palsies of the abducens, trigeminal, oculomotor, and trochlear cranial nerve on the right side and left-sided hemiparesis. A significant increase in tumor size to 50 × 54 × 45 mm with anterior, parasellar, and infratentorial extension was seen (Fig. 2). Again, repeat decompression surgery was done. Two months after surgery, there was no improvement in clinical features and repeat imaging suggested an increased size of the tumor by 30%, to approximately 86 × 68 × 75 mm. Nine years after initial presentation, the patient had an episode of aspiration pneumonia and died.

## Discussion

Radiation-induced tumors were initially described by Cahan et al in 1948. They also described the prerequisites for a tumor to be classified as a radiation-induced sarcoma [5]. The modified Cahan criteria state that (1) the presence of nonmalignancy or malignancy of a different histological type before irradiation, (2) development of sarcoma within or adjacent to the area of the radiation beam, (3) a latent period of at least 3 years between irradiation and diagnosis of secondary tumor, and (4) histological diagnosis of sarcoma, can be classified as radiation-induced sarcoma [5].

Our patient fulfilled the criteria for a radiation-induced sarcoma with a highly malignant tumor on histopathology. Radiation-induced sarcomas after functional pituitary tumors, especially Cushing disease, are rarely reported. One of the case reports revealed a high-grade osteoblastic osteosarcoma 30 years after treatment for Cushing disease with transsphenoidal resection and external beam radiotherapy [6]. In our case, there was a lag period of approximately 5 years before the appearance of a second highly undifferentiated, malignant, histologically distinct tumor. The cellular origin of this relatively undifferentiated tumor cannot be determined with certainty. However, the interlacing sarcomatous and adenomatous components resulting from distinct positive immunohistochemistry may indicate that the sarcomatous component may be derived from the preexisting pituitary adenoma.

A hormonally functional pituitary tumor is not itself expected to be associated with an increased risk of secondary malignancy, except in the case of GH-secreting tumors and those with a hereditary cancer syndrome. Although not proven, immunosuppression from hypercortisolism in Cushing disease has been proposed as a contributor to secondary tumor development [7]. Other mechanisms causing increased risk of secondary malignancy can be double-stranded DNA damage and genomic instability caused by ionizing radiation and germline mutations in tumor suppressor genes such as TP53 and Rb [7].

Radiation-induced intracranial tumors were studied in a multicenter, retrospective cohort of 4292 patients with pituitary adenoma or craniopharyngioma. Radiotherapy exposure was associated with an increased risk of a second brain tumor with a rate ratio of 2.18 (95% CI, 1.31-3.62, *P* < .0001). The cumulative probability of a second brain tumor was 4% for the irradiated patients and 2.1% for the controls at 20 years [7]. In another study including 426 patients irradiated for pituitary adenoma between 1962 and 1994, the cumulative risk of second brain tumors was 2.0% (CI, 0.9-4.4) at 10 years and 2.4% (95% CI, 1.2-5.0) at 20 years. The relative risk of a second brain tumor compared with the incidence in the normal population is 10.5 (95% CI, 4.3-16.7) [8].

The incidence of radiation-induced sarcomas has been estimated at 0.03% to 0.3% of patients who have undergone radiation therapy. The risk of radiation-induced sarcomas increases with field size and dose. In a systemic review and analysis of 180 cases of radiation-induced intracranial sarcomas, the average dose of radiation delivered was 51.4 ± 18.6 Gy and latent period of sarcoma onset was 12.4 ± 8.6 years. A total of 49 cases were developed after radiation treatment of pituitary adenomas (27.2%). The median overall survival time for all patients with sarcoma was 11 months, with a 5-year survival rate of 14.3% [9].

Our patient received approximately 50 Gy twice through fractionated radiotherapy, resulting in larger field size and significantly higher dose than one would expect with a modern stereotactic treatment. Such a high dose of radiation is indeed a risk factor for secondary malignancy. In our patient, in a period of 2 months, there was already >30% tumor growth after recent repeat decompression surgery.

The risk of secondary malignancy is thought to be much lower with stereotactic radiosurgery than conventional external beam radiation therapy, with an estimated cumulative incidence of 0.045% over 10 years (95% CI, 0.00-0.34) [10]. However, long-term follow-up data for patients receiving stereotactic radiation therapy are shorter and thus definitive conclusions cannot be made at this stage.

Our case highlights a rare but devastating long-term complication of pituitary tumor irradiation after Cushing disease. The limited response to various available treatment options defines the aggressive nature of radiation-induced malignancy.

## Learning Points

- The occurrence of a second neoplasm constitutes possibly one of the most adverse and rare complication after radiotherapy.
- The incidence of radiation-induced sarcomas has been estimated at 0.03% to 0.3% of patients, but cases after Cushing disease are rarely reported.
- Patients often present with advanced disease unresponsive to various treatment modalities because of aggressive clinical course.
- New modalities with stereotactic radiosurgery and proton beam therapy are to be reviewed closely for risk assessment of secondary tumor.

## Data Availability

Data sharing is not applicable to this article as no data sets were generated or analyzed during the current study.
